# Natural Phyto-Bioactive Compounds for the Treatment of Type 2 Diabetes: Inflammation as a Target

**DOI:** 10.3390/nu8080461

**Published:** 2016-08-04

**Authors:** Sivapragasam Gothai, Palanivel Ganesan, Shin-Young Park, Sharida Fakurazi, Dong-Kug Choi, Palanisamy Arulselvan

**Affiliations:** 1 Laboratory of Vaccines and Immunotherapeutics, Institute of Bioscience, Universiti Putra Malaysia, Serdang 43400, Malaysia; gothai_86@yahoo.com (S.G.); sharida.fakurazi@gmail.com (S.F.); 2 Nanotechnology Research Center and Department of Applied Life Science, College of Biomedical and Health Science, Konkuk University, Chungju 380-701, Korea; palanivel67@gmail.com (P.G.); choidk@kku.ac.kr (D.-K.C.); 3 Department of Biotechnology, College of Biomedical and Health Science, Konkuk University, Chungju 380-701, Korea; ifresha@nate.com; 4 Department of Human Anatomy, Faculty of Medicine and Health Sciences, Universiti Putra Malaysia, Serdang 43400, Malaysia

**Keywords:** type 2 diabetes mellitus, insulin resistance, polyphenols, inflammatory mediators, diabetic complications

## Abstract

Diabetes is a metabolic, endocrine disorder which is characterized by hyperglycemia and glucose intolerance due to insulin resistance. Extensive research has confirmed that inflammation is closely involved in the pathogenesis of diabetes and its complications. Patients with diabetes display typical features of an inflammatory process characterized by the presence of cytokines, immune cell infiltration, impaired function and tissue destruction. Numerous anti-diabetic drugs are often prescribed to diabetic patients, to reduce the risk of diabetes through modulation of inflammation. However, those anti-diabetic drugs are often not successful as a result of side effects; therefore, researchers are searching for efficient natural therapeutic targets with less or no side effects. Natural products’ derived bioactive molecules have been proven to improve insulin resistance and associated complications through suppression of inflammatory signaling pathways. In this review article, we described the extraction, isolation and identification of bioactive compounds and its molecular mechanisms in the prevention of diabetes associated complications.

## 1. Introduction

Presently, there are more than 415 million people affected by diabetes mellitus worldwide, according to the International Diabetes Federation, and this figure is projected to rise to over 642 million or more by 2040. Around 90% of diabetic patients in the worldwide are diagnosed with type 2 diabetes mellitus (T2DM). The cost of health care related to diabetes and its secondary complications continues to expand and is a massive economic burden for afflicted diabetic patients and particularly developing countries (Diabetes Atlas, 7th edition, International Diabetes Federation, 2015). T2DM represents a major global health issue and incidence of diseases increases with various genetic and other associated factors such as age, obesity, stress, diet, ethnicity, lack of exercise and inflammation. The burden of type 2 diabetes and its major complications are rising worldwide [1].

Inflammation has been recognized as a key player in the pathophysiology of both type 1 and type 2 diabetes and its secondary complications [2]. Chronic low-grade inflammation and an activation of the various immune reactions are particularly involved in the pathogenesis of obesity-linked insulin resistance and type 2 diabetes. Activated inflammatory markers are major factors to initiate and develop diabetes-associated complications including retinopathy, nephropathy, neuropathy, ischemic heart disease, peripheral vascular disease, and cerebrovascular disease, etc. [3,4]. Therefore, targeting the inflammation and its signaling pathways may be an active target to prevent/manage diabetes mellitus and its associated complications. Existing therapeutic drugs used for diabetes, which increase various secondary complications including cardiovascular disease, kidney failure, liver injury, dizziness, mental disorders, weight gain, and skin diseases [5].

Natural products and its derived active compounds may be achievable alternatives for the treatment of type 2 diabetes and its complications without any adverse effects. There are a huge number of active medicinal plants and its natural bioactive molecules that have already reported the therapeutic nature against diabetes [6]. Several medicinal plants have been used since ancient times to manage and prevent diabetes and associated conditions [7]. 

Looking into the list of drugs approved within the last decades demonstrates that plant ingredients are still of importance in drug discovery. Plant compounds have been shown to confer some protection against the pathology of diabetes mellitus through the attenuation of inflammatory mediators. Therefore, this paper intends to review the most salient recent reports on the anti-inflammatory associated diabetes mellitus properties of phytochemicals and the molecular mechanisms underlying these properties. 

## 2. Inflammation Status in Diabetes Mellitus

Insulin resistance or T2DM has been well-defined as a state of universal inflammation condition involving both innate and adaptive immunity [3]. Preclinical and clinical studies have demonstrated that various anti-inflammatory agents can improve blood glucose level and pancreatic beta cell function in T2DM [8,9]. Thus, inflammatory associated pathways are one of the principal pathways in the pathogenesis of T2DM and its complications.

T2DM are associated with increased expressions of complete markers of chronic inflammation and the primary molecular link between inflammation and T2DM are macrophage mediators, tumor necrosis factor-α (TNF α), interleukin-1β (IL-1β) and interleukin-6 (IL-6). Elevated amounts of these pro-inflammatory cytokines have been confirmed in diabetes mellitus [10]. The trend of diabetic patients to have higher amounts of inflammation status has serious consequences that contribute to both microvascular and macrovascular complications [11]. Among both types of complications, macrovascular complications are critical for human life, namely, cardiovascular disease and also around 80% of diabetic patients die from coronary artery diseases and its related complications [12]. 

Monocyte chemoattractant protein-1 (MCP-1) is a key chemokine produced by mainly adipocytes upon recruitment of macrophages and endothelial cells [13]. The augmented release of circulating MCP-1 during adiposity, promotes the expression of pro-inflammatory cytokines thereby, it impairs the inflammation associated with type 2 diabetes. Adiponectin is one of the most important anti-inflammatory cytokines secreted by white adipose tissue. The level of adiponectin mainly reduces in obesity, and inflammation associated type 2 diabetic conditions; meanwhile, the increased level of adiponectin was observed during type 1 diabetes mellitus (T1DM) [14]. 

Toll-like receptors (TLRs) are playing a central role in innate immunity by their capability to sense pathogens across the pathogen-associated molecular patterns (PAMPs) and to notice tissue injury through the danger-associated molecular patterns (DAMPs) [15]. Among different types of TLRS, TLRs 1,2,4,5,6, and 11 are plasma membrane proteins, while TLRs 3,7,8 and 9 exist in intracellular compartments. Various factors including microbial constituents provoke the activation of the TLR (excluding for TLR3) signaling through a MyD88 (myeloid differentiation factor)-dependent pathway, principal to the activation of the signaling transcription factor NF-κB and the production of inflammatory mediators. These TLR signaling pathways might activate through the production of numerous factors including endogenous HMGB1 (High-Mobility Group Box 1), and advanced glycation end products (AGEs) [15,16]. 

HMGB1 is also one of the ligands for activation for TLR2 and 4, and it has also been elevated in an experimental diabetic animal model. It was previously recognized as one of the transcription factor regulator, and it was later confirmed as a cytokine produced through cell damage and immune cells like macrophages, thus HMGB1 actively stimulates a NF-κB signaling cascade [17,18]. 

Advanced glycation end products (AGEs), which were principally believed as oxidative derivatives, due to from diabetic hyperglycemia conditions, and these are gradually realized as a possible risk factor for pancreatic islet β-cell injury and type 2 diabetes. These AGEs significantly elevated the inflammatory markers as well as oxidative markers in diabetic conditions; meanwhile, it impairs the production and action of insulin [19]. 

Numerous preclinical and clinical studies have clearly established that adipose tissue, livers, muscles and pancreases are major sites of inflammation of the occurrence of obesity and T2DM [2]. An infiltration of macrophages into adipose tissue, liver, muscle and pancreas are seen in obesity and diabetes-induced animal models and in obese human individuals with T2DM. Macrophages are essential for the production of pro-inflammatory cytokines [20], including TNFα, IL-6, IL-1β and other inflammatory mediators. These inflammatory mediators act in an autocrine and paracrine way to stimulate insulin resistance by interfering with insulin signaling in peripheral tissues through activation of various inflammatory associated pathways such as nuclear factor-kappa B (NF-κB) and c-JUN N-terminal kinase (JNK) pathways [21,22]. These pathways are responsible for promoting the tissue inflammation in obesity and diabetic condition. 

At the molecular level, signaling pathway, NF-κB is the inflammation principal switch that controls the synthesis of numerous active proteins series such as IκB, IL-1β, IL-1, and TNF-α for the activation and maintenance of the inflamed state. In obesity condition, it stimulates the NF-κB activation and associated pathways in adipose tissue, livers, and pancreases, thus promoting insulin resistance and T2DM. Researchers have proven that inflammation is not only a marker, but it is also a mediator of disease that was confirmed earlier. 

The immune cells’ macrophages can be categorized into two distinct subtypes: the “classically activated macrophages” phenotype, termed M1, which produce major pro-inflammatory cytokines including TNF-α, IL-6, IL-1β and the “alternatively activated macrophages” phenotype, termed M2, which produces major anti-inflammatory cytokines, IL-10 [20]. Furthermore, macrophage infiltration in to adipose tissue and obesity causes a phenotypic switch from the M2 to M1 phenotype, connecting with insulin resistance in animals and humans [23]. The M1 phenotype of macrophages can alter insulin signaling pathways and adipogenesis in adipocytes while M2 macrophages appear to safeguard against obesity-induced insulin resistance [20]. 

Increased expression/production of TNFα in adipose tissue was observed in obese individuals, and it is playing the vital role in obesity-induced insulin resistance [24]. Previous findings have been confirmed a specific up-regulation of inflammatory genes and an over-production of numerous pro-inflammatory cytokines and chemokines in inflamed adipose tissue [2]. Furthermore, improvement in insulin sensitivity induced by weight loss was accompanied by a reduction in the expression of multiple pro-inflammatory genes [2,25]; hence, inflammation in adipose tissue was considered as a crucial consequence leading to T2DM and its complications. 

**Figure 1:**
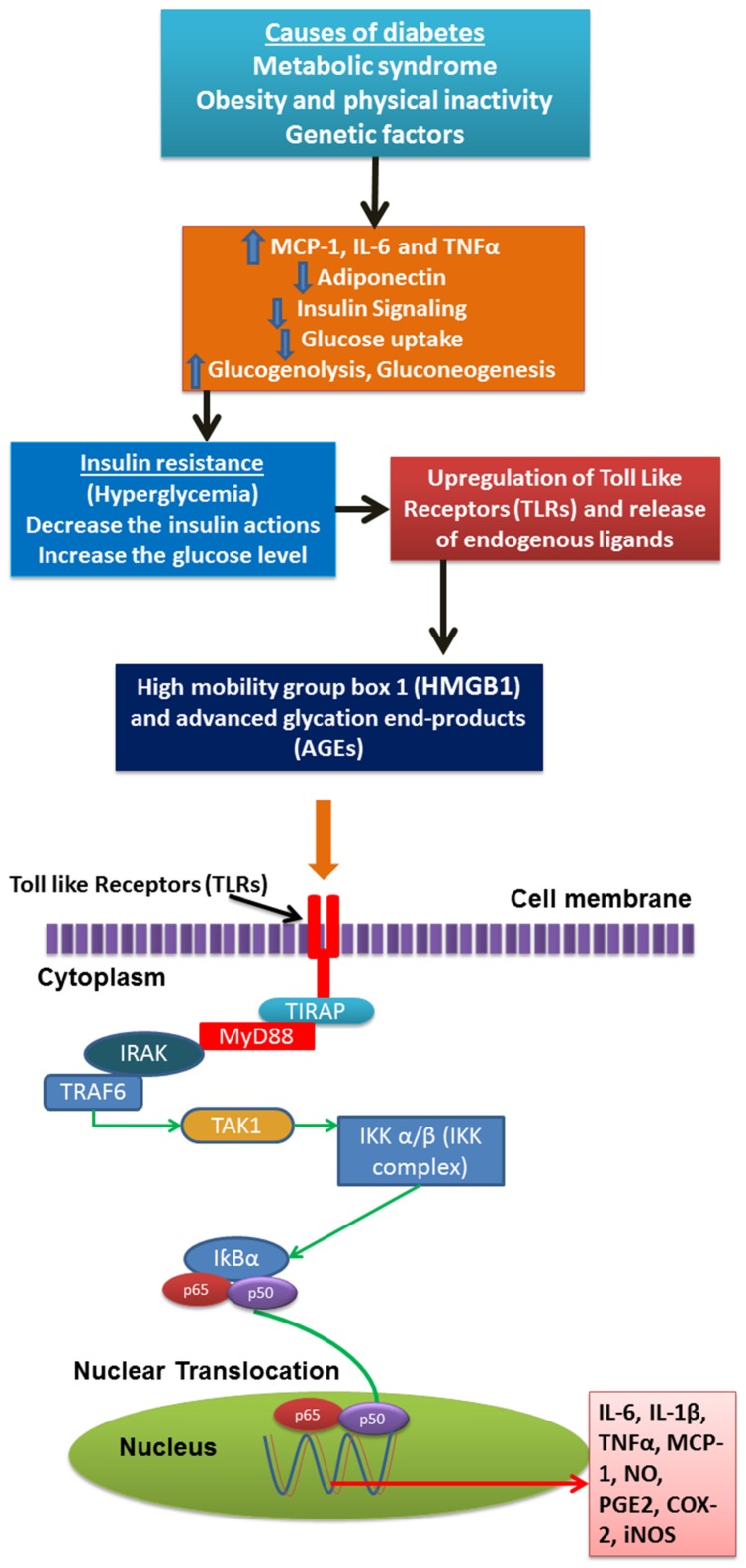
Activation of inflammatory pathway and inflammatory mediators in diabetic condition.

In the conclusion, previous investigations reveal a complex interaction between cells of innate and adaptive immunity system and the equilibrium among these immune cells turns out to be essential for the homeostasis and control of tissue inflammation in obesity and T2DM (Figure 1). 

## 3. Anti-Inflammatory Based Therapeutics for Diabetes

Several therapeutic interventions are very effective in reducing acute and chronic inflammation and improving diabetes and its complications via indirect or pleiotropic mechanisms. Issues that reduce the inflammation (particularly, key inflammatory markers such as pro-inflammatory mediators TNFα, IL-6, IL-1β and CRP) could offer a vital public health tool to reduce the burden of diabetes and associated complications including cardiovascular diseases in the general population. The probability of regulating innate immunity-related inflammation as an important experimental approach for the management/prevention of T2DM is based on findings that investigated the therapeutic efficacy of anti-inflammatory agents [26]. 

Nowadays, the key therapeutic agents to treat T2DM and its complications, sulfonylureas, metformin, and insulin-sensitizing glitazones all improve metabolic control and lead to control of various circulating inflammation mediators through innate immunity-related signaling pathways. Sulfonylureas and metformin are main drugs to prevent the T2DM, and sulfonylureas increase insulin production from pancreatic β-cells, while metformin suppresses glucose production in the liver and meanwhile increases insulin sensitivity in peripheral tissues [27]. Glitazones, another anti-diabetic drug, binds to peroxisome proliferator-activated receptors (PPARs), beginning a transcriptional activity that leads to improved insulin action through reducing the secretion of inflammatory markers. Consequently, glitazones reduced levels of CRP, PAI-1, TNF-α and other inflammatory markers. These drugs showed better anti-diabetic nature and also have the comparable anti-inflammatory potential [26,28].

Other therapeutic approaches for T2DM that would act as principals in the inflammatory system have been proposed in the form of salicylates, an anti-inflammatory therapeutic that inhibits IκB kinase (IKK), and also lowering the glucose level through improvement of beta cell function [29]. Various well established non-steroidal anti-inflammatory drugs (NSAIDs) and cyclooxygenase inhibitors (e.g., ibuprofen, naproxen) are able to improve glucose-mediated insulin release, glucose tolerance, and reduce the insulin resistance in diabetic patients [30,31]. 

In clinical studies, treatment with NSAIDs enhanced many biochemical indices such as blood glucose level, glucose uptake, insulin clearance, CRP, lipid profile associated with obesity, and T2DM [32]. Though these findings support the notion that inflammation plays a key role in T2DM and its complications, attenuating inflammation as a strategy for disease prevention in a public health setting will demand a markedly different perspective. In this case, an approach that can be introduced into the population with the minimal side effects and the maximal therapeutic result should be adopted. In this connection, natural products based therapeutic approach would be a better opportunity to treat inflammatory associated chronic diseases like T2DM and its complications, since natural products derived agents are generally safer, lower cost and more highly available in the world.

## 4. Natural Phyto Bioactive Compounds Role in Anti-Diabetics

Natural phyto bioactive compounds are currently more in demand than the synthetic medicines for the treatment of diabetes owing to the rich availability, efficacy and fewer side effects [33,34]. The higher amount of these phyto bioactive compounds rich foods can be consumed on the daily basis that can enhance the antidiabetic activities. It also performed well in various traditional medicines includes Indian Ayurvedic and Chinese traditional medicines and showed enhanced bioactivity of those phyto compounds [35,36]. These phyto bioactive compounds rich extracts either as a single or combination of the multiple extracts showed enhanced anti-diabetic activities. These combination extract therapy aids in multiple actions of those bioactive compounds by single therapies thereby enhance the beneficiary activities, which, in turn, reduces the drug load to the patients. Among the drugs approved for the anti-diabetic activities in the last 10 years, 49% derived from the plant origin. Overall, 1200 phyto bioactive compound rich plants were reported for their better antidiabetic activities, among them 400 phyto bioactive rich plant extracts showed type 2 anti-diabetic activities [34,35,36]. Phyto bioactive compounds include saponin, myrcelin, flavonoids, pectin, and glucosides rich in various parts of the plants showed enhanced antidiabetic activities. The antidiabetic activities of these phytocompounds can be varied based on mechanisms of their actions for lowering the glucose including glucose absorption, target insulin resistance and pancreatic functions.

Phyto bioactive compounds rich in extracts of safflower and Japanese kelp showed higher suppress-glucosidase activity, thereby regulating the glucose absorption in the gut [37,38]. In another study, Inulin, the soluble fiber showed regulation of GLp-1 homeostasis [39]. Further regulation of insulin resistance was reported by various phyto bioactive compounds rich plants such as Dioscorea polysaccharides, blueberry anthocyanins, cinnamon and fenugreek seeds [40,41,42,43]. In addition, several phyto extracts showed combinational effect of these bioactivities and its include chili peppers, bitter melon, ginseng, turmeric and tea extracts [44,45,46,47,48,49]. Therefore, the combinational intake of these foods or synergistic efficacy of these phyto compounds will be a future research area in the diabetic disease models. Even though these phyto compounds showed multiple beneficial effects in various in vitro studies, their extraction method limits their actions in their bioactivity of these compounds. A few of those novel technologies used in the extraction of those bioactive compounds and their roles in the anti-diabetic activity are discussed below.

## 5. Extraction, Identification and Characterization of Bioactive Compounds from Natural Products

World Health Organization (WHO) reported that more than 25,000 medicinally valuable plants exist in different countries including in both developing and developed countries. The principal steps to exploit the bioactive phyto-compounds from natural resources are solvent extraction, bioassays, isolation, and characterization, toxicological, preclinical and clinical investigation of bioactive phyto-compound. 

We need to establish the different appropriate methods to investigate the biological properties of phyto compounds from the natural products, and these biological assays are required to identify and isolate the bioactive compounds present in the extract. The entire in vitro biological assay is very reliable and a simple method to get to know the biological activities of extracts, and it may differ based on the targeted diseases like anti-cancer, anti-diabetes, anti-inflammation, anti-malarial, anti-microbial and toxicity studies. 

### 5.1. Extraction

Extraction from the natural products is the first step to screen the biological activities of extracts and identify the bioactive compounds for further isolation, identification and characterization. These steps include washing and drying the raw materials, and blending to obtain the powder and using a different solvent system to extract the crude extract to investigate its biological activities [50,51].

Appropriate extraction methods should be used to ensure that potential bioactive constituents are not lost, distorted or destroyed during the preparation of the crude extract from samples [40]. The selection of a suitable solvent system basically depends on the physical nature of the bioactive compound being targeted as various solvent systems are available to extract/identify the bioactive compound from natural products. The hydrophilic compound extraction, mainly of polar solvents including methanol, ethanol or ethyl-acetate, and for lipophilic compounds extraction, dichloromethane or mixtures of dichloromethane/methanol in the ratio of 1:1, was used to identify the compounds [52]. 

Recently, various modern extraction techniques were used by natural products based researchers, which include solid-phase micro-extraction, supercritical-fluid extraction, pressurized-liquid extraction, microwave-assisted extraction, and surfactant-mediated techniques. These modern extraction techniques have a lot of advantages including the decrease in organic solvent consumption and in sample stability, removal of unwanted samples clean-up and solvent concentration steps before chromatographic separation, improvement in extraction productivity, selectivity, kinetics of extraction. The exhaustive extraction method is also one of the most efficient extraction methods, and it is mostly carried out with different solvent systems of increasing polarity in order to extract the most bioactive components with significant biological properties. Based on the advantages of these modern techniques, researchers have used these techniques to identify the bioactive compounds more efficiently [53].

Effective identification and isolation of bioactive compounds from natural products are mainly subject to the type of solvent used in the extraction methods. The solvent selection will also depend on the polarity of targeted bioactive compounds to be extracted from natural products [54]. Moreover, the molecular similarity between solvent and solute, mass transfer, use of co-solvent method, toxicity nature, ease of solvent evaporation at low temperature, stability, and inability to cause the extract to composite or dissociate. The choice of solvent selection was affected by other factors such as the amount of phytocompounds to be extracted, the frequency of extraction, the variety of phytocompounds to be extracted, ease of successive handling of the crude extracts and cost effectiveness.

### 5.2. Novel Technologies Used in Extraction of Phyto Based Antidiabetic Compounds

Extraction of the bioactive compounds from the anti-diabetic plants showed a crucial step in determining the antidiabetic activity of the phyto extracts with higher yield and greater potency of those extracts. For the higher yield extraction of the compounds, several factors need to be considered in conventional technologies such as the type of sample, solvent, mixture of those solvents [55]. In order to overcome several difficulties in conventional technologies includes higher use of organic solvents and sample degradation, several modern technologies serve as an alternative for the extraction of those bioactive compounds from antidiabetic plants. Those technologies include but are not limited to ultrasound extraction, microwave-assisted extraction and supercritical extraction. These technologies overcome the conventional technologies by reducing organic solvents usages, higher yield and elimination of unwanted compounds from the extract and showed the greater antidiabetic efficiency of those phyto bioactive compounds [53].

### 5.3. Ultra Sound Extraction of Phyto Compounds from Anti-Diabetic Plants

Ultra sound extraction technology is one of the most modern technologies frequently used in the extraction of phyto bioactive compounds from antidiabetic plants with enhanced anti-diabetic bioactivities [56]. This technology enhances the extraction yield with many highly stable bioactive compounds from the antidiabetic plants by the enhanced cell penetrating effect of solvents and breaking of the intramolecular forces through high intensity sound waves [56,57]. The main advantage of these techniques is that they can able to extract the maximum bioactive compounds with limited raw material and shorter time. Recently, polysaccharides from mulberry fruits were extracted using ultrasound technology at an extraction time of about 75 min, produced an extraction yield of about 3.13%, and showed higher anti-glycemic activity by stronger α-glucosidase inhibition activity [58]. Similarly, higher extraction yield of total polyphenol was also obtained by ultrasound assisted extraction of guava leaves extract with higher anti-hyperglycemic activity than acarbose [56]. Likewise, the higher extraction yield of α-glucosidase enzyme was also obtained in the guava leaves with maximum antihyperglycemic activities. Similarly anthocyanins extraction yield increases using ultrasound assisted extraction of bilberry, blackberry and mulberry fruits. The extraction yield of those anthocyanins reached a maximum of up to 2800 mg/L. These extracts showed enhanced anti hyperglycemic activity in the diabetic rats [57]. In another study, the antidiabetic activity of the *Pterocarpus marsupium* Roxb. Heartwood can be enhanced with ultrasound extraction with the higher protection of the phyto bioactive compounds than other conventional methods in alloxan induced antidiabetic rats [59]. From all of these studies, it was confirmed that extraction yield of the phyto bioactive compounds can be enhanced through the ultrasound extraction technologies, and it also opens ways for the researchers to use these non-conventional technologies for the higher extraction of the bioactive compounds from the anti-diabetics plants.

### 5.4. Microwave Assisted Extraction of Phyto Compounds from Antidiabetic Plants

Microwave assisted extraction is another novel technology currently used in the extraction of phyto bioactive compounds from various medicinal plants like ginger, tea, mango, sapotta [60,61,62,63,64,65] and other herbs proven to have antidiabetic activities. The microwave technology is highly used in the extraction of phyto bioactive compounds with lesser degradation of those compounds, higher extraction yield and cost effectiveness than other conventional technologies. Recently, antidiabetic activity of microwave assisted extraction of *Solanum nigrum* leaves was studied and found that higher extraction yield of *Solanum nigrum* leaves extract were obtained using microwave assisted extraction with a yield of 64%. The antidiabetic activities of those extracts were found to be dose dependent. Higher and medium doses were found to have a greater antidiabetic effect. In addition, for the extraction of the bioactive compounds, drying using the microwave technology also enhances the extraction yield of the phyto bioactive compounds. Recently, leaves of *Aquilaria subintegra* and *Aquilaria malaccensis* were dried using microwaves and were found to increase the yield of bioactive compounds with enhancing antidiabetic activity [66]. In addition to the extraction and antidiabetic study of the phyto bioactive compounds, several phyto bioactive compounds were studied for the effective extraction using microwave assisted extraction technology with higher extraction yield [67,68]. However, their efficacy in various diabetic models is still limited. This will provide a new research scope about the role of microwave technology in extraction and bioactivity against various diabetic models in near future.

### 5.5. Supercritical Fluid Extraction of Bioactive Compounds from Antidiabetic Plants

Supercritical fluid extraction technology is one of the novel technologies for the extraction of phyto bioactive compounds from the antidiabetic plants [69,70,71,72] with possible application of those ingredients in the developments of the functional foods and pharmaceutical industries. This technology has several advantages over conventional technologies like higher yield, and multiple compounds can be extracted from this technology, and it can ultimately lead to multiple health benefits of those products developed using these extracts. In addition, this technology has several other advantages including the low viscosity of supercritical fluid that leads to multiple extractions, less solvent residue, and faster extraction process [72]. Recently, various non-polar constituents from *Toona sinensis* leaves were extracted using supercritical fluid extraction technology and found 24 different active compounds. The major compound in those extractions was that of phytol which possesses greater antidiabetic activity along with the prevention of hepatosteatosis [72]. The same research group also checked for the commercial essential oils obtained by a supercritical extraction process showed greater antidiabetic activity owing to the higher non polar constituents. Similarly, bixin, a colored pigment that possesses anti-hyperglycemic activity, was obtained by a supercritical extraction process with higher yield [73]. Several bioactive compounds can also be effectively extracted from the anti-diabetic plants such as ginseng, turmeric, but their active role in the diabetic model of supercritical extracted compounds is still yet to be elucidated.

Overall, modern extraction processes such as ultrasounds, microwaves, and supercritical fluid extraction process can efficiently extract various phyto bioactive compounds with higher extraction yield, faster process, and lesser thermal degradation, possessing multiple benefits in anti-diabetic activity. However, certain modern equipment is cost effective such as the supercritical fluid extraction process, requires much space, and needs to be considered in future. Table 1 illustrates the modern extraction techniques used for the preparation of bioactive compounds from medicinal plants. 

### 5.6. Purification of Bioactive Compounds

Purification of bioactive compounds from crude extracts is an important task, as purified compounds are much more therapeutically effective. Recent modern techniques have created numerous ways for the purification and large-scale production of several bioactive compounds [77]. The plant crude extracts generally occur as a combination of various categories of bioactive compounds with different polarities, hence different levels of chromatographic separations are used to purify the bioactive compounds including Thin Layer Chromatography (TLC), Column Chromatography and High Performance Liquid Chromatography (HPLC). These chromatographic techniques are still used to purify/isolate the bioactive compounds due to various advantages including convenience, and availability of the variety of stationary phases for separation of active phytochemicals [75,76]. 

Mainly, TLC has been used to the fraction of different components from natural products and this technique shortens the process of isolation and identification of bioactive compound [74]. HPLC is one of the important widely used purification techniques for the isolation of natural products followed by TLC and other chromatographic techniques [78]. Presently, HPLC is gaining popularity among other analytical chromatographic techniques as the key choice for phytocompound fingerprinting. The bioactive molecules are often existing only as a minor component in the crude extract and the determination ability of HPLC is ideally suited to the quick processing of multicomponent natural products based samples on both an analytical and preparative scale [79].

Apart from HPLC, there are other alternative analytical methods employed to identify phytocompounds, among which is the diode array detector (DAD) coupled with a mass spectrometer (MS), liquid chromatography and gas chromatography coupled with mass spectrometry (LC/MS and GC/MS) [79,80]. These alternative analytical methods provide abundant useful information for structural elucidation and identification of the bioactive compounds when tandem mass spectrometry (MS) is applied. Thus, the combination of HPLC with MS facilitates prompt and more accurate identification of phytocompounds from natural products sample. 

LC-NMR is the combination of chromatographic separation and structural elucidation of an unknown compound and mixture. It is the one of the most powerful and a time saving method to isolate the structures of interested molecules [81]. The current introduction of a pulsed field gradient technique in high resolution NMR, together with a three-dimensional technique enhances application in structure elucidation and molecular weight information. These new hyphenated techniques are valuable in the regions of pharmacokinetics, toxicity studies, drug metabolism and drug discovery process [82].

### 5.7. Structural Characterization of Purified Compounds

The isolated and purified phytocompound needs to be structurally determined to analyze the chemical properties of the compound. This process involves accumulating information from a wide range of spectroscopic techniques, including UV-visible spectroscopy, Infra-Red (IR), and NMR, which provides a certain clue regarding the structure of the isolated/purified molecule. Characteristics of the isolated phyto-compound can be analyzed by UV-visible spectroscopy and the IR spectroscopy technique has proven to be a precious tool for the characterization and identification of compounds and/or its functional groups (chemical bonds) present in an unidentified mixture of the natural products extract [83]. The identification of functional groups in a compound may be detected using IR by analyzing the different bonds present. 

NMR spectroscopy is the most unswerving tool for the elucidation of molecular structures. An NMR microscope defines the number and types of nuclei in an organic molecule, describing the individual chemical environment and their interconnection. Swotting the molecule of interest with NMR spectroscopy permits the determination of differences in the magnetic properties of the various magnetic nuclei present and atoms are present in neighboring groups [84,85]. 

## 6. Natural Anti-Diabetic/Inflammatory Bioactive Compounds and Their Mechanisms of Action

The chemical substances from living organisms are identified as natural compounds. The primary sources of natural compounds are plants. Plant synthesize diverse groups of natural compounds, commonly referred to as secondary metabolites and their function in plants is now attracting attention because of their use as dyes, glues, oils, waxes, flavoring agents, drugs and perfumes, and they are noticed as potential sources of natural drugs, antibiotics, insecticides and herbicides, etc. [7,86]. Currently, the role of some secondary metabolites as protective dietary elements has become a progressively vital area in human nutrition based research. Evidence affirms that modest long-term intakes can have favorable impacts on the incidence of many chronic inflammatory associated diseases/disorders, including T2DM (Table 2) [7,87]. 

**Figure 2:**
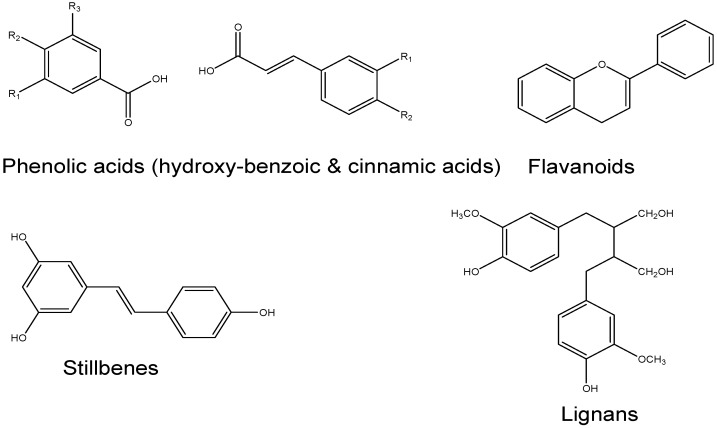
Chemical structures of the different classes of polyphenols.

Based on their biosynthetic origins, plant secondary metabolites can be divided into three major groups such as (i) phenolic compounds; (ii) terpenoids and (iii) nitrogen-containing alkaloids and sulphur-containing compounds. Compared to another group of secondary metabolites, phenolic compounds are largely responsible for beneficial effects on human health [88], and these naturally occurring compounds are found largely in fruits, vegetables, cereals and beverages [89]. The content of the polyphenols in a plant is greatly affected by environmental factors like sun exposure, soil types, rainfall and stress, etc. [90,91]. Phenolics that are not soluble are found in cell walls, while soluble phenolics are present within the plant cell vacuoles (Figure 2) [92]. Phenols are classified into different groups as a function of the number of phenol rings in the structure and their main classes include phenolic acids, flavonoids, stilbenes and lignans.

Two classes of phenolic acids are derivatives of benzoic acid and cinnamic acid. The hydroxybenzoic acid content of edible plants is generally very low, with the exception of certain red fruits, black radishes, and onions. Because these hydroxybenzoic acids, both free and esterified, are found in only a few plants eaten by humans, they have not been extensively studied and are not currently considered to be of great nutritional interest. The hydroxycinnamic acids are more common than are the hydroxybenzoic acids and consist chiefly of *p*-coumaric, caffeic, ferulic, and sinapic acids. These acids are rarely found in the free form, except in processed food that has undergone freezing, sterilization, or fermentation [93]. 

Favonoids comprise the most studied group of polyphenols. Flavonoids may be divided into six subclasses: flavonols, flavones, flavanones, flavanols, anthocyanins and isoflavones based on the variation in the type of heterocycle involved. They are typically found in the form of glycosides and sometimes as acylglycosides, while acylated, methylated and sulfate molecules are less frequent and in lower concentrations. They are water-soluble and accumulate in cell vacuoles [88].

Stilbenes are a subgroup of non-flavonoid polyphenols with two phenyl moieties connected by a two-carbon methylene bridge and are found in low quantities in the human diet. One of these is resveratrol, and the protective effect of this molecule is unlikely at normal nutritional intakes [94].

Lignins fall under the subgroup of non-flavonoid polyphenols. They are diphenolic compounds that contain a 2,3-dibenzylbutane structure that is formed by the dimerization of two cinnamic acid residues. Several lignans, such as secoisolariciresinol, are the richest dietary source of linseed. They are considered to be phytoestrogens [95].

Diabetes has been recognized as an oxidative stress based disorder caused by an imbalance between the cellular production of reactive oxygen species and the counteracting antioxidant mechanisms by body’s natural antioxidants [126]. Studies have suggested that oxidative stress are enacted in systemic inflammation, endothelial dysfunction, impaired secretion of pancreatic β-cells and glucose utilization in peripheral tissues that lead to long-term secondary complications [127]. Growing evidence from epidemiological studies suggests a positive association between reduction in the incidence diabetes and the consumption of a diet rich in phenols [128]. Several biological beneficial properties have been documented for dietary phenols including antioxidant [129], anti-allergic [130], anti-viral [131], anti-microbial [132], anti-proliferative [133], anti-carcinogenic [134], free radical scavenging [135] and regulation of cell cycle arrest [136]. 

Phenol-rich foods increase plasma antioxidant capacity, and this incidence may be explained by acceptance of electron from reactive oxygen species (ROS), thus forming relatively stable phenoxyl radicals (Clifford). ROS are considered to be a toxic byproduct, pose a threat to cells by causing peroxidation of lipids, oxidation of proteins, and damage to nucleic acids, enzyme inhibition, activation of a programmed cell death (PCD) pathway, and ultimately lead to death of the cells [137]. Phenols, therefore, protect cell constituents against oxidative damage and limit the risk of various degenerative diseases associated with oxidative stress. Likewise, inflammation and stress are both responsible for the pathogenesis of T2DM, suggesting the potential importance of antioxidants and anti-inflammatory alternatives [138]. The phenolic compounds with potent anti-oxidant activity are capable of exhibiting anti-inflammatory activity, with the potential to prevent DM and its complications. The current review is an attempt to provide a description of various plants’ derived phenolic compounds currently used for treatment, and inhibition of inflammatory pathways that are important in diabetic prevention strategies.

**Figure 3:**
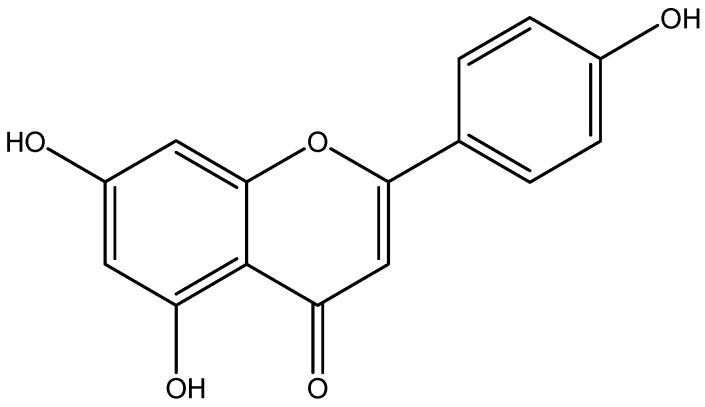
Chemical structure of Apigenin.

Apigenin is a natural flavonoid abundantly present in common fruits and vegetables (Figure 3) [139]. Apigenin as a therapeutic agent for various inflammatory diseases inhibits TNF-α and IL-1β-induced activation of NF-κB via ERK1/2 activation. Apigenin attenuates production of pro-inflammatory cytokines such as IL-6, IL-1β, and TNF-α through modulating multiple intracellular signaling pathways in macrophages, which ameliorated hyperglycemic and improved antioxidants via oxidative stress-related signaling [97].

**Figure 4:**
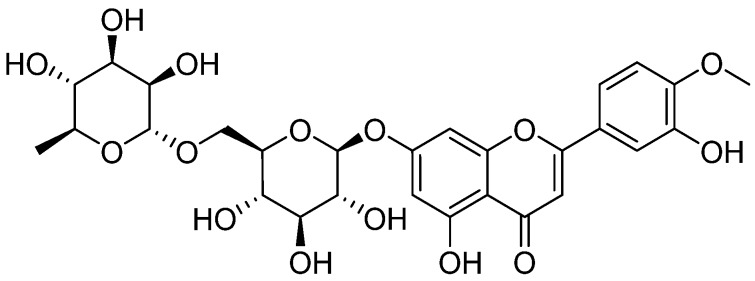
Chemical structure of Diosmin.

Diosmin is a naturally occurring flavonoid, abundant in the pericarp of various citrus fruits (Figure 4) [140]. Excessive production of free fatty acids (hyperglycaemia) has previously been shown to cause inflammation leading to mitochondrial DNA damage and pancreatic cell malfunctioning [141]. Antioxidant supplementation by diosmin suppressed diabetes induced ROS resulting in deactivation of NF-κB associated pro-inflammatory chemokines and cytokines such as macrophage chemotactic protein (MCP-1), tumor necrosis factor (TNF-α), and interleukins (IL-1β and 6) [98].

**Figure 5:**
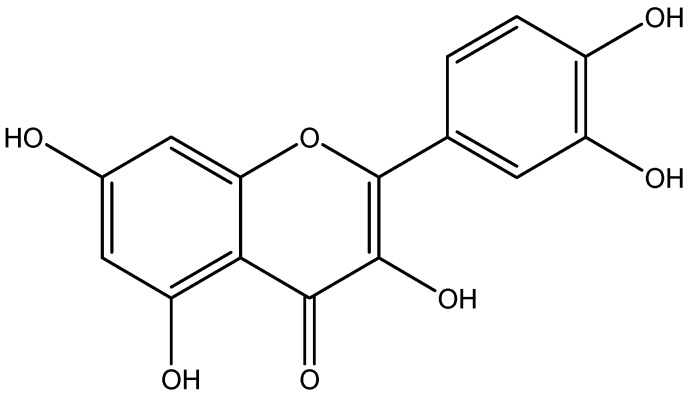
Chemical structure of Quercetin.

Quercetin is found in a great variety of food, including vegetables, tea, apples, grapevines, berries, broccoli, red onions and capers (Figure 5) [142]. Inflammatory mediators can activate a number of receptors, which subsequently result in pancreatic β-cell dysfunction, insulin signaling impairment, endothelial dysfunction and altered vascular flow that lead to diabetic vascular complications [143]. CRP, a marker of systemic inflammation, and markers of endothelial dysfunction have been reported in both type 1 and type 2 diabetic patients [144]. Quercetin administrations protect against diabetes-induced exaggerated vasoconstriction. These effects resulted from the reduction in serum level of both TNF-α and CRP and inhibition of aortic NF-kβ in both models of diabetes [100].

**Figure 6:**
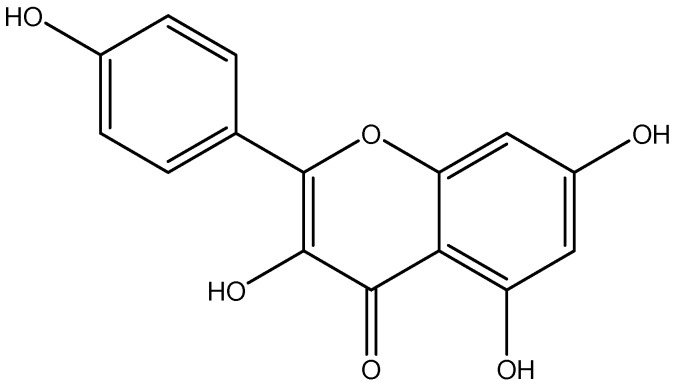
Chemical structure of Kaempferol.

Kaempferol is a natural flavonol, relatively abundant in grapefruit, tea cruciferous vegetables and some edible berries (Figure 6) [145]. Previous findings showed that antioxidant content of Kaempferol reduced IL-1β, TNF-α [101], and kaempferol was also reported to significantly decrease the fasting blood glucose and improve insulin resistance. Anti-inflammatory and anti-diabetic effects of kaempferol are mediated by through AMPK activation [102].

**Figure 7:**
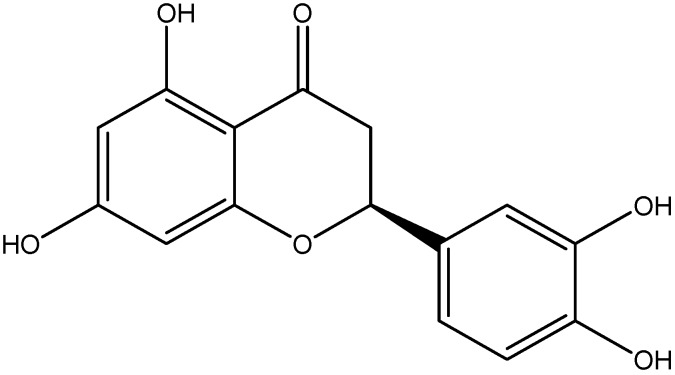
Chemical structure of Eriodictyol.

Eriodictyol is a bitter-masking flavanone extracted from lemon, Indian beech and rose hips. Eriodictyol has been reported to possess anti-inflammatory properties, by significantly lower retinal TNF-α, intercellular adhesion molecule 1 (ICAM-1), vascular endothelial growth factor (VEGF), and endothelial NOS (eNOS) (Figure 7) [104]. Supplementation with eriodictyol suppressed diabetes with upregulation of mRNA expression of PPARγ2 and adipocyte-specific fatty acid-binding protein as well as the protein levels of PPARγ2 in differentiated 3T3-L1 adipocytes. Furthermore, eriodictyol reactivated Akt in HepG2 cells with HG-induced insulin resistance [103].

**Figure 8:**
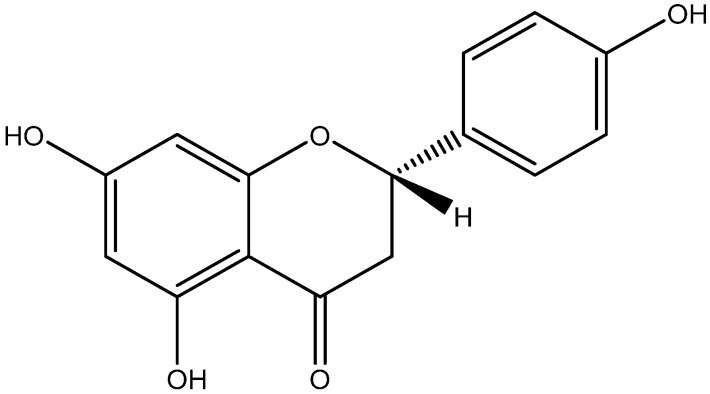
Chemical structure of Naringenin.

Naringenin is a flavonoid found in fruits including grapefruit, oranges, and tomatoes with robust antioxidant potential (Figure 8) [146]. Naringenin was also found to prevent reactivity in diabetic via upregulation of both 5′ AMPK. Stimulation of innate immunity by high blood glucose, induce inflammation and lead to type 2 diabetes [147]. Naringenin administration upregulates the activation of the AMPK pathway and then increases glucose tolerance and insulin sensitivity [106].

**Figure 9:**
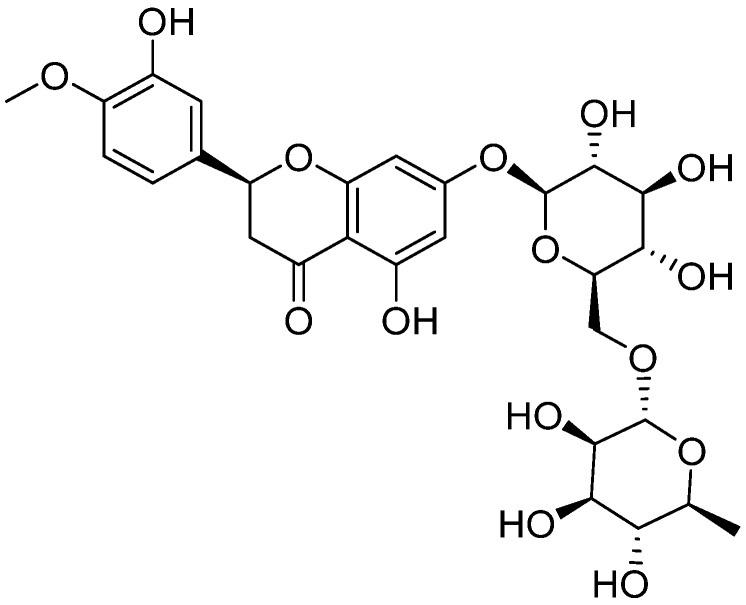
Chemical structure of Hesperidin.

Hesperidin is a flavanone glycoside found in citrus fruits, orange, and lemon (Figure 9) [148]. Hesperidin attenuates the diabetic condition through control over hyperglycemia and hyperlipidemia by downregulation of free radical generation, and the release of pro-inflammatory cytokines. Reduced oxidative stress by hesperidin due to strong antioxidant capacity was also found to be helpful in the prevention of damage caused by oxygen free radicals of cellular organelles and its related enzymes and development of insulin resistance [108].

**Figure 10:**
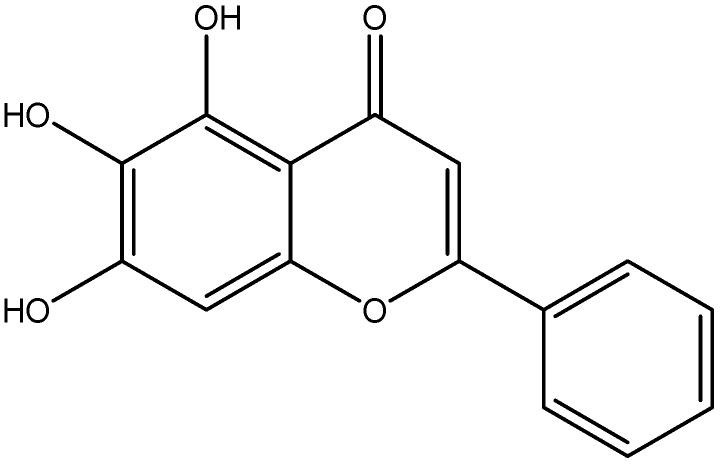
Chemical structure of Baicalein.

Baicalein, a flavonoid, was originally isolated from the roots of *Scutellaria baicalensis*, *Scutellaria lateriflora* and fruits of *Oroxylum indicum* (Figure 10) [149]. Baicalein was reported to suppress the activation of NF-κB, and decrease expression of iNOS and TGF-β1, which support its anti-inflammatory property [110]. Baicalein displayed significant improvement in hyperglycemia, glucose tolerance, and insulin levels. Mechanism of its action was by upregulation of AMPK and its related signal pathway, a regulator of metabolic homeostasis involving inflammation and oxidative stress. Activated AMPK could abolish inflammation through the MAPK signaling pathway. Activated AMPK could attenuate insulin resistance by phosphorylating IRS-1, AKT and dephosphorylate ERK, JNK and NF-κB. It also suppresses fatty acid synthesis, gluconeogenesis and increases mitochondrial β-oxidation [111].

**Figure 11:**
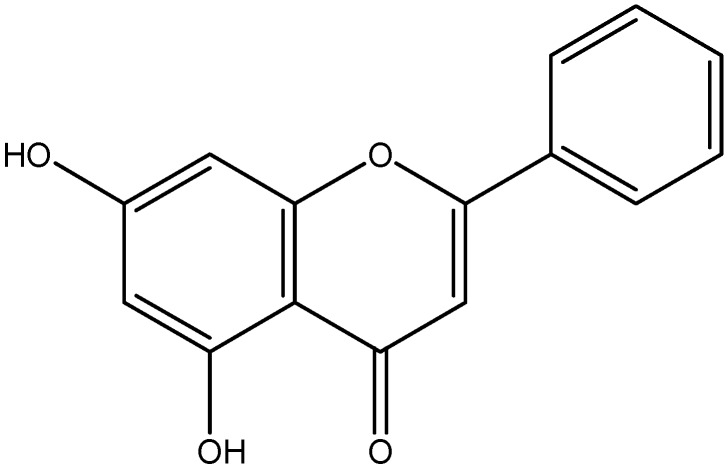
Chemical structure of Chrysin.

Chrysin is a naturally occurring flavonoid and is found in honey, propolis, fruits, vegetables, beverages and medicinal plants such as *Passiflora caerulea*, *Pelargonium peltatum* and *Tilia tomentosa* Moench (Figure 11) [150]. According to Ahad et al. [112], treatment with chrysin reduced the serum levels of pro-inflammatory cytokines, IL-1β and IL-6. Consequently, chrysin prevents the development of diabetes through anti-inflammatory effects, specifically targeting the TNF-α pathway.

**Figure 12:**
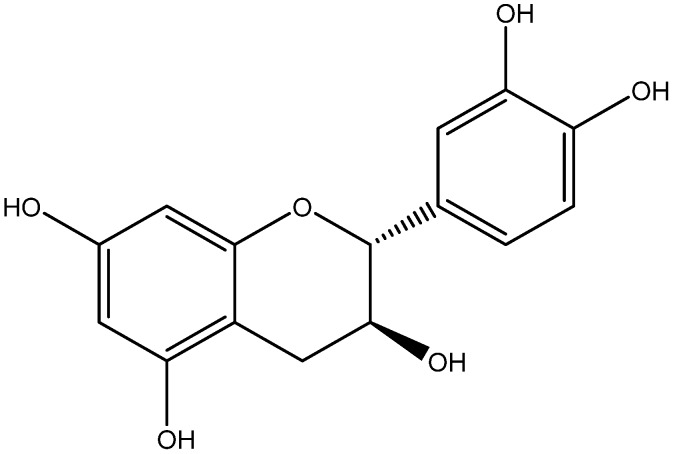
Catechin is a crystalline four molecule flavonoid compound (C_15_H_14_O_6_).

Catechin is a flavonol, and high concentrations of catechin can be found in grapes, berries, apples, dark chocolate, ginger, tea and cocoa (Figure 12) [151]. Chronic nutrient surplus and excessive energy balance activate stress in adipose tissue leading to stimulation of the innate immunity defense [152], and provoke inflammation to protect the organism against cellular damage and pathogen invasion. Chronic inflammation can aggravate diseases like obesity, type 2 diabetes, and atherosclerosis [147]. Catechin effectively suppresses the activation of NF-κB system through inhibition of the secretion of pro-inflammatory cytokines (TNF-α and IL-6), specifically into the adipose tissue [114,153,154]. The inhibitory effects of Catechin on the oxidative-inflammatory loop contribute to its therapeutic efficacies against diabetes. 

**Figure 13:**
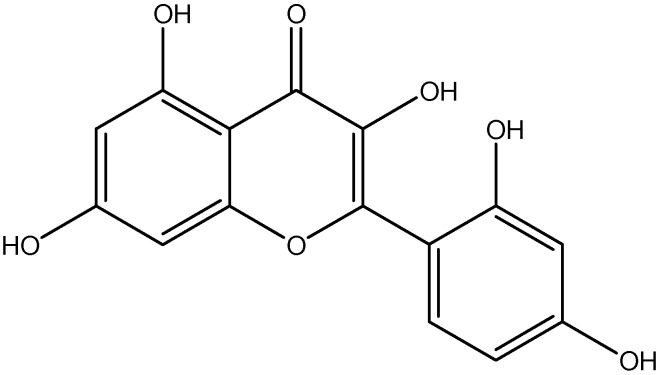
Chemical structure of Morin.

Morin, a major active component of traditional medicinal herbs from almond, guajava (common guava) and wine (Figure 13) [155]. A study demonstrates that management of diabetes with morin reduced the elevation of inflammatory cytokines IL-1β, IL-6 and TNF-α via a SphK1/S1P signaling pathway. This activity supports its anti-inflammatory property and possible beneficial effects in diabetes [116]. Subsequently, treatment with morin expressively abridged the blood glucose, glucose metabolic enzymes and improved the production of insulin levels in the diabetes model [117].

**Figure 14:**
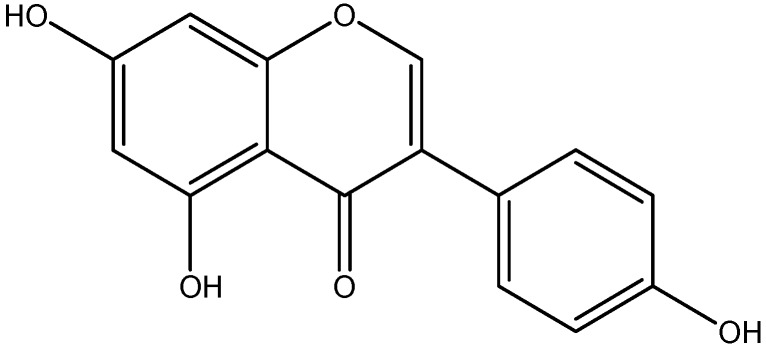
Chemical structure of Genistein.

Genisteins are mainly isolated from soybeans and other legumes, such as chickpeas, contain small amounts of genistein (Figure 14) [156]. Diabetic retinopathy is affiliate with microglial activation and increased levels of inflammatory cytokines (TNF-α). This inflammatory signal involves the activation of tyrosine kinase and its subsequent events, ERK and p38 MAPK pathways. These effects of diabetes in retinas were reduced by intervention treatment with genistein. Genistein, a tyrosine kinase inhibitor, represses the release of TNF-α and significantly inhibits ERK and P38 phosphorylation in activated microglial cells [118,157,158].

**Figure 15:**
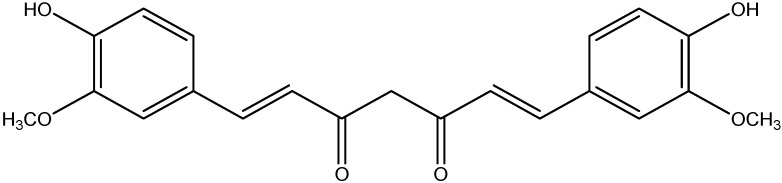
Chemical structure of Curcumin.

Curcumin is the major active component of turmeric, and it is one of the polyphenol compounds that exhibits antioxidant, anti-inflammatory, anti-tumorigenic, and antimicrobial properties (Figure 15). Hyperglycemia-induced ROS can stimulate NF-κB activation which then causes the increase in vascular adhesion molecule expression (ICAM-1), which plays a central role in diabetic vascular inflammation [159]. Overexpression of their adhesive capability (ICAM-1) is considered as the main event in the development of atherosclerosis associated diabetes [160]. Curcumin supplementation ameliorates diabetic vascular inflammation through the decrease in ROS overproduction and ICAM-1 expressions [120].

**Figure 16:**
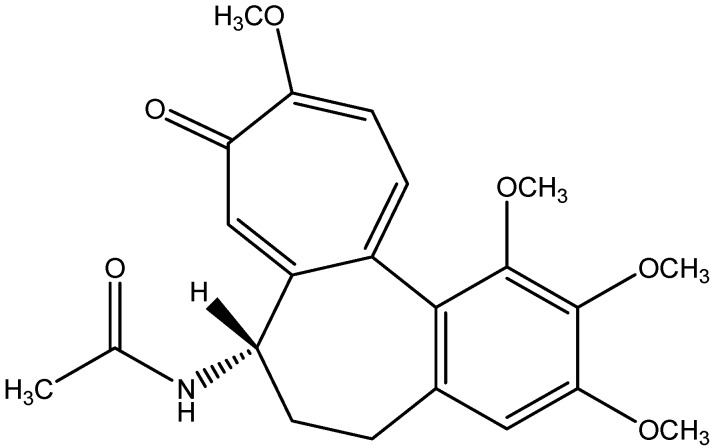
Chemical structure of Colchicine.

Colchicine was originally extracted from the *Colchicum autumnale* (Colchicaceae) plant and also contained in the corms of *Colchicum luteum*, and the seeds of *Gloriosa superba* (Figure 16). Colchicine is traditionally considered the staple therapy for inflammatory diseases such as gout and pericarditis [161]. Elevated infiltration of inflammatory cells in renal tubulointerstitium is commonly seen in diabetic nephropathy patients [162]. Chemokines and adhesion molecules such as monocyte chemotactic protein (MCP)-1 and intercellular adhesion molecule (ICAM-1) expression are increased in diabetes nephropathy. Colchicine supplementation mitigates inflammatory cell infiltration in diabetic nephropathy by inhibiting MCP-1 and ICAM-1 expression [121].

**Figure 17:**
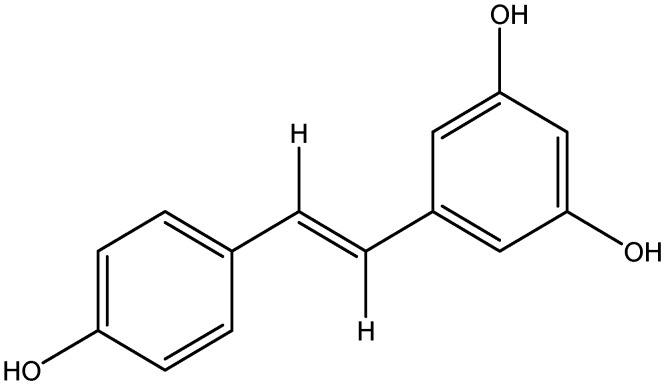
Chemical structure of Resveratrol.

Resveratrol is a type of natural phenol compound, and a high concentration of resveratrol is found in the skin and seeds of grapes, peanuts and ground nuts (Figure 17) [161]. Diabetes is usually associated with inflammation, and an excess level of glucose is shunted via alternative pathways, which, in turn, leads to an increase in TGF-β1 and NF-κB (inflammatory mediators). Upregulation of NF-κB is convoyed by the COX-2 [163] enzyme, and it is responsible for the production of prostaglandins, inflammatory mediators produced by activated macrophages/monocytes as well as microglia in the neuroinflammatory diseases [164]. Administration of resveratrol significantly ameliorated diabetes inflammation [123] by acting as a potent scavenger of ROS, which deters lipid peroxidation, which is induced by oxidative stress [165]. In addition, resveratrol attenuates the activation of immune cells and release of pro-inflammatory mediators through the inhibition of NF-κB, followed by downregulated COX-2 gene expression in the diabetic model [123].

**Figure 18:**
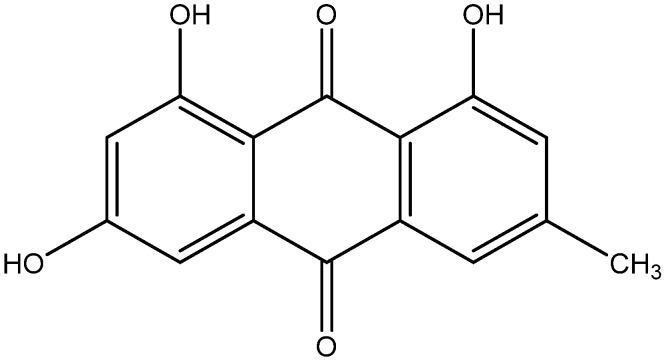
Chemical structure of Emodin.

Emodin is a major active component from Aloe vera, kiwi fruits, lettuce and banana. Emodin exerts anti-inflammatory effect via suppressing the activation of NF-κB in human umbilical vein endothelial cells (Figure 18) [166]. In addition, Emodin proved to have antidiabetic properties via inhibiting the degradation of IκB, an inhibitory subunit of NF-κB. When added, emodin also downmodulated adhesion molecules like ICAM-1, and VCAM-1 contains NF-κB binding sites in their promoter region in endothelial cells that could reduce the impact of type 2 diabetes [124,125].

## 7. Phytochemicals Bioavailability and Their Effect on Human Metabolism

Bioavailability is defined as substances obtained from ingested materials that reach circulatory systems for further delivery into designated tissues so that the beneficial compounds are biologically available for exerting healthy functions. The normal routes of dietary phytochemicals thus include ingestion, digestions, and transport across gastrointestinal epithelium prior to circulatory vessels. Phytochemicals are located inside vacuoles and cell walls of plant cells. Most cell wall materials are indigestible by human enzymic systems. Therefore, digestibility of the phytochemicals is of great interest or reveals how the phytochemicals can affect human health and fight or prevent diseases [167].

### 7.1. Digestibility of Phytochemicals

The processing factors (food texture, e.g., heat, temperature, or pressure application) can impinge on the bioaccessibility (fraction of a compound that is released from the matrix and potentially available for further uptake and absorption) of bioactivity [168]. The elimination of a natural barrier of the cell wall yields a better release of phytocompounds. For example, more phenolic is obtained from tomato juice than those from dried and fresh tomato, indicating that the natural barrier of the cell wall has been eliminated. In contrast, chlorogenic acid is present in fresh products, but it gradually disappears in juice. This could be caused by the different extractability due to different matrices of the products or by chemical changes due to processing and digestion environments [169].

Compounds that are free from cell wall materials show clearer responses during gastrointestinal digestion. Phenolic stability is strongly affected by pH. For example, flavonols and proanthocyanidins remain intact, but they may also be broken down when pH is sufficiently low in the stomach. pH higher than 7.4 is unfavorable for phenolics, and the effects of high pH are worsened by lengthy exposures. This results from oxidation further into diketones and other degradation products. The number of –OH groups in benzene rings of simple phenolics can also be critical clues for phenolic stability [170]. 

### 7.2. Absorption of Phytochemicals

Most absorptive tissues are comprised of epithelial cells that protect the human body from hazardous components in ingested foods. There is no well-established molecular form of absorbed substances in the gastrointestinal tract, i.e., whether they are absorbed intact or as metabolites. The influence of enzyme concentrations, solubility, pH, and time of digestion all play a role and influence bioaccessibility and absorption [171].

For example, the release of carotenoids increases significantly in intestinal digestion where bile extract and pancreatic secretions exist. Consecutive gastrointestinal digestions do not help with higher release of carotenoids. This is more likely due to insufficient emulsifier–water ratios to provide emulsification of carotenoids that are fat-soluble [172].

### 7.3. Bioavailability of Phytochemicals

Phytochemicals bioavailability is strongly dependent on cell wall compositions of the food matrices they originate from, the structural chemistry of the phytochemicals, history of processing, and the individual human gastrointestinal system. This factor greatly alters the structure and profile and thus the potential bioactivity of many plant compounds that are not absorbed in the small intestine. These complexities determine the dietary phytochemicals plan that should be recommended in order to reach biologically-safe active dosages [173]. 

## 8. Conclusions

Inflammation emerges to contribute to the pathogenesis of T2DM and its secondary complications, particularly in cardiovascular disease. Various researchers have investigated the underlying mechanisms that initiate inflammation and link it to insulin resistance and associated complications. These investigations may provide new opportunities for treating type 2 diabetic patients and its complications. Most of the anti-diabetic drugs have the ability to control the glucose level and improve insulin secretion through anti-inflammatory mechanisms, but they have few undesirable effects based on the preclinical and clinical investigations. The current investigations have suggested that natural products derived bioactive compounds act as a therapeutic tool in chronic inflammatory diseases. Mostly, polyphenols appear to be significant metabolic modulators by virtue of their capability to influence various cellular and molecular pathway targets, which have been proven as potential targets for the polyphenolic group of compounds. Nevertheless, clinical use of natural products based active compounds has not yet been investigated properly through intracellular signaling pathways. Further research will be needed to fully explain the cellular and molecular mechanisms of actions of natural products’ derived compounds and their analogues in several physiological processes, in order to yield essential insights into their prophylactic and therapeutic uses.

## Figures and Tables

**Table 1:** Novel extraction methods of extracting bioactive compounds from anti-diabetic plants.

Serial No.	Extraction Methods	Phyto Bioactive Compounds	Plant Parts	Antidiabetic Activities	Reference
1	Ultrasound assisted extraction	Polysaccharides	Mulberry fruits	α-glucosidase inhibition	[62]
		Polyphenols	Guava leaves	Anti-hyperglycemic	[60]
		Anthocyanins	Berry fruits	Anti-hyperglycemic	[61]
		Crude extract	Heart woods	Anti-diabetic	[63]
2	Microwave assisted extraction	Crude extracts	Night shade leaves	Anti-diabetic	[74]
		Dried leaves extracts	Aquilaria leaves	Anti-diabetic	[70]
3	Supercritical fluid extraction	Phytol	Toona sinensis leaves	Antidiabetic	[75]
		Bixin	Annatoo seeds	Anti-hyperglycemic	[76]

**Table 2:** Classification of bioactive compounds and their major plant sources with therapeutic targets for inflammation associated diabetes.

Class	Compounds	Plant Sources	Mechanism of Actions	Reference
Flavone	Apigenin	Parsley	1. Activation of ERK1/2 2. Attenuates the production of pro-inflammatory cytokines such as IL-6, IL-1β, and TNF-α	[96,97]
		Celery		
		Rosemary		
		Oregano		
		Thyme		
		Basil		
		Coriander		
		Chamomile		
		Cloves		
	Diosmin	Lemon	1. Deactivation of NF-κB targets 2. Suppression of monocyte chemoattractant protein-1 (MCP-1), tumor necrosis factor (TNF-α), and interleukins (IL-1β and 6)	[98]
		Orange		
		Buddha fingers		
Flavonol	Quercetin	Capers	1. Inhibition of NF-κB system 2. Reduction in serum level of both TNF-α and CRP	[99,100]
		Onions		
		Cranberries		
		Blueberrie		
		Chokeberris		
	Kaempferol	Tomatoes	1. AMPK activation 2. Decrease the fasting blood glucose, and improved insulin resistance	[101,102]
		Green Tea		
		Potatoes		
		Broccoli		
		Brussels		
		Sprouts		
		Squash		
	Eriodictyol	Lemons	1. Suppress the activation of NF-κB system 2. Reduce TNF-α, intercellular adhesion molecule 1 (ICAM-1), vascular endothelial growth factor (VEGF), and endothelial NOS (eNOS)	[103,104]
		Mountain balm		
Flavanone	Naringenin	Grapefruit	1. Activation of AMPK and suppression of NF-κB pathways 2. Increases the glucose tolerance and insulin sensitivity	[105,106]
		oranges		
		tomatoes		
	Hesperetin	LemonOrange	1. Suppress the activation of NF-κB system 2. Down-regulation of pro-inflammatory cytokines and oxidative stress markers	[107,108]
		Peppermint		
		Tangerine		
	Baicalein	ParsleyCellery	1. Activation of AMPK pathway 2. Suppresses fatty acid synthesis, gluconeogenesis and increases the mitochondrial β-oxidation	[109,110,111]
		Capsicum		
		Pepper		
	Chrysin	Skullcap	1. Suppression of TNF-α production and activation of NF-κB activation 2. Reduce the serum levels of pro-inflammatory cytokines, IL-1β and IL-6	[112]
		Honey		
Flavanol	Catechin	Green tea	Suppress the activation of NF-κB system through the inhibiton of pro-inflammatory cytkines productions	[113,114]
		Chocholate		
		Beans		
		Cherry		
	Morin	Indian guava	1. Modulation of SphK1/S1P signaling pathway 2. Reduce the elevation of inflammatory cytokines IL-1β, IL-6 and TNF-α	[115,116,117]
		Green tea extract		
		Almond		
Isoflavonoid	Genistein 4′,5,7-OH	Soy flour	1. Represses the release of TNF-α production 2. Inhibits the activation of ERK and P38 phosphorylation	[118]
		Soy milk		
		Soy beans		
Phenolic acid	Curcumin	*Turmeric*	1. Suppression of ICAM-1 expressions & ROS 2. Improves Vascular inflammation Inhibits MCP-1 & ICAM-1 expressions	[119,120]
		*Curry powder*		
		*Mango Ginger*		
	Colchicine	Saffron	1. Mitigates inflammatory cell infiltration 2. Suppression of MCP-1 and ICAM-1expression	[121]
		Colchicum		
Stilbene	Resveratrol	Grapes	1. Suppress the activation of NF-κB signaling pathway 2. Downregulates the COX-2 gene expression which increase the release of pro-inflammatory mediators	[122,123]
		Wine		
		Grape		
		Peanuts		
		Cocoa		
		Berries		
	Emodin	Japanese knotweed	1. Suppress the activation of NF-κB system 2. Down-modulated the adhesion molecules including ICAM-1, and VCAM-1.	[124,125]
		Rhubarb		
		Buckthorn		
